# Supplementary material for: Hairpin trimer transition state of amyloid fibril
Source: Nat Commun. 2024 Mar 29;15:2756. doi: 10.1038/s41467-024-46446-x (PMC10980705; doi:10.1038/s41467-024-46446-x)
Supplement: Supplementary file 1 — Supplementary Information [file 41467_2024_46446_MOESM1_ESM.pdf]

SUPPLEMENTARY INFORMATION

Supplementary Note 1. Tau derived mutant peptides

Sequence of tau microtubule-binding repeat domain is shown in Supplementary Fig. 1. The P301L mutant of the 17-residue long fragment peptide between R2 and R3 subdomains and the corresponding one between R1 and R3 domains are also given. The 3R tau isoform lacks the second repeat domain, and so R1 is connected directly to R3. This gives rise to a modified sequence in the N-terminal side of the fragment peptide. There are 3 residues that are different when 3R derived peptide is compared to that of 4R. These are positions 295, 297, and 300. They are E, L, and Q in 3R form while D, I, and V in 4R form respectively. Some of the mutants studied here are just single-point mutants of the 4R fragment peptide, like P301L, P301S, G303V, S305N, etc. On the other hand, some mutants are chimeric single, double, and triple mutations from the 3R fragment peptide to that of the 4R peptide. These mutants are labeled as "ED" to represent E295D, "LI" for L297I, "QV" for Q300V, and their possible double and triple combinations as we go from non-aggregating P301L mutant of 3R fragment peptide to fast aggregating P301L mutant of 4R peptide, as shown with the bottom left arrow in Supplementary Fig. 1

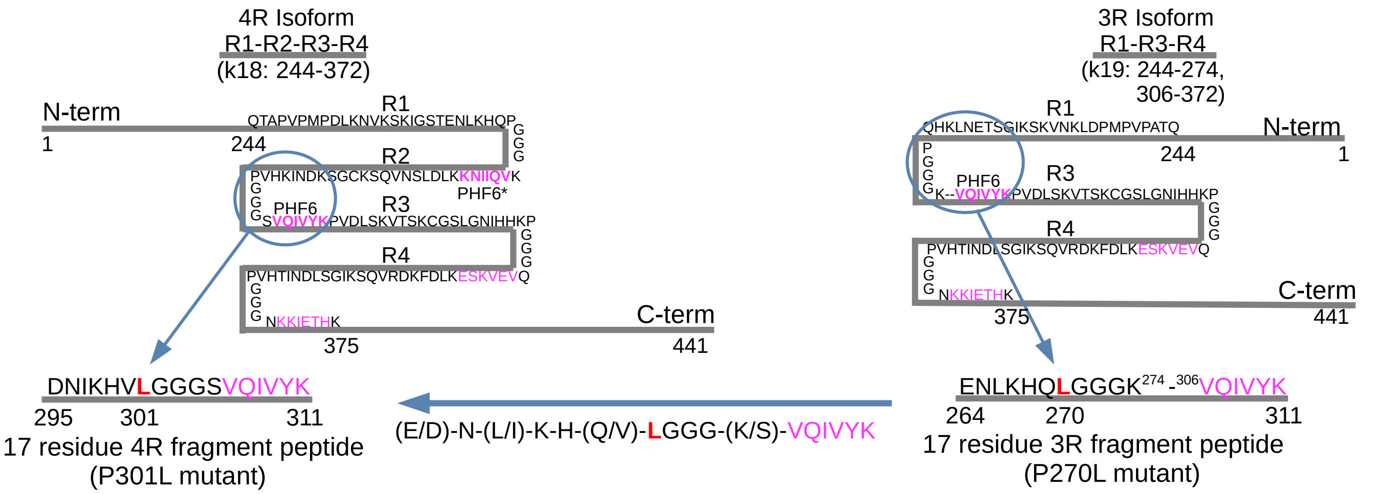

Supplementary Figure 1. *Protein model system. The repeat domain of tau protein, which forms the tau fibril core, and the P301L mutants of the 17 residue-long R2R3 and R1R3 fragment peptides. Mutations at four residues; 264E to 295D (DE), 266L to 297I (LI), 269Q to 300V (QV), and 274K to 305S (KS) that converts R1R3 fragment to R2R3 is shown with a blue left arrow. k18 and k19 constructs are as defined in Ref. 46 in the Main Text*

Supplementary Note 2. Verification of REMD/Amber96 method on known structures

Two different de-novo designed short peptides with known NMR structures were chosen. The first one is a stable, monomeric beta-hairpin (SWTWENGKWTWK, 1le1.pdb) [S1], while the second one is a alpha-helical short peptide (SAAEAYAKRIAEAMAKG, 2i9m.pdb). Five independent folding simulations for each peptide starting from a linear structure, with the same exact REMD/Amber96/GBSA protocol as described in the methods section was performed. Then, final conformations were clustered using 3 Å cut-off distance with the GROMOS clustering method [S2]. The median structures of the first largest clusters perfectly match the NMR folds, with 1.2 Å RMSD and the same hydrogen bond registry for beta-hairpin, and 2.4 Å RMSD and the same hydrogen bonding pattern for alpha-helical peptide (Supplementary Fig. 2) For the hairpin peptide, the largest cluster has a very dominant probability of 71.9%, while other hairpin-looking partial folds have only a few percent probabilities in the ensemble. For the helical peptide, a complete alpha helix was obtained with 32.9% probability in the first cluster, while the second and third clusters are partial folds of the same NMR helix with 12.9% and 7.2 % probabilities respectively. Overall, 82% of the conformations do have some sort of helical characters while those with beta-sheets are less than 3%. In fact, with a loose cut-off value

of 5 Å, 69.4% of the conformations turn out to be near-complete alpha helical folds where the median structure has an RMSD of 2.7 Åfrom the NMR helix. These results confirm that REMD/GBSA/Amber96 combination is able to correctly produce both alpha-helical and beta-sheet folds for short peptides, and there is no bias toward any secondary structure.

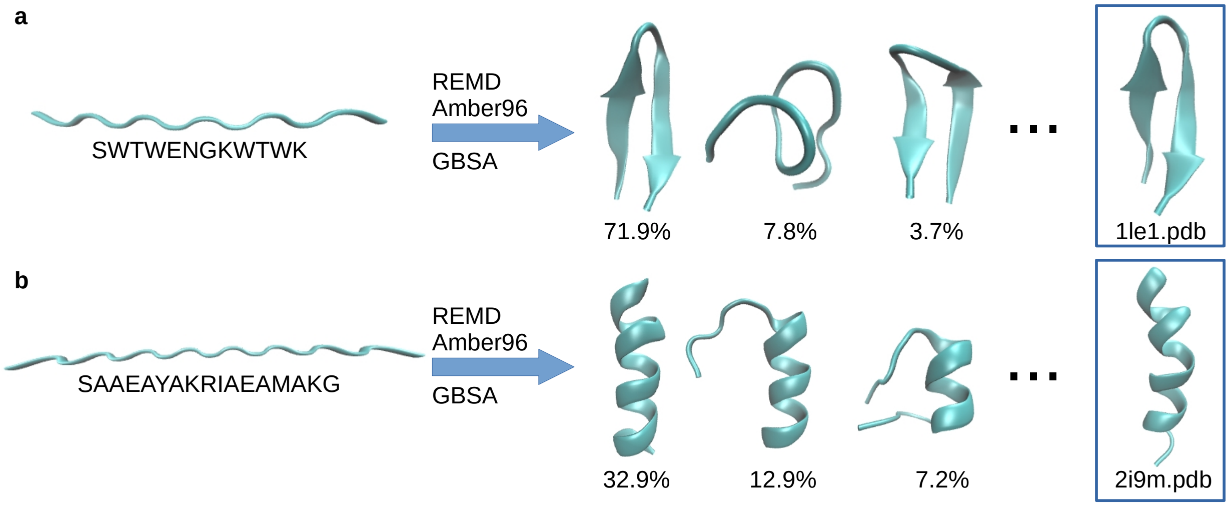

Supplementary Figure 2. *Verification of the simulation method. REMD/Amber96/GBSA is able to reproduce both beta-hairpin and alpha-helical NMR structures*

Supplementary Note 3. Simulation Result Details

REMD sampling at the chosen six temperatures showed dominant energy overlaps with good average exchange probabilities around 0.35 to 0.40 (Supplementary Fig.3)

a

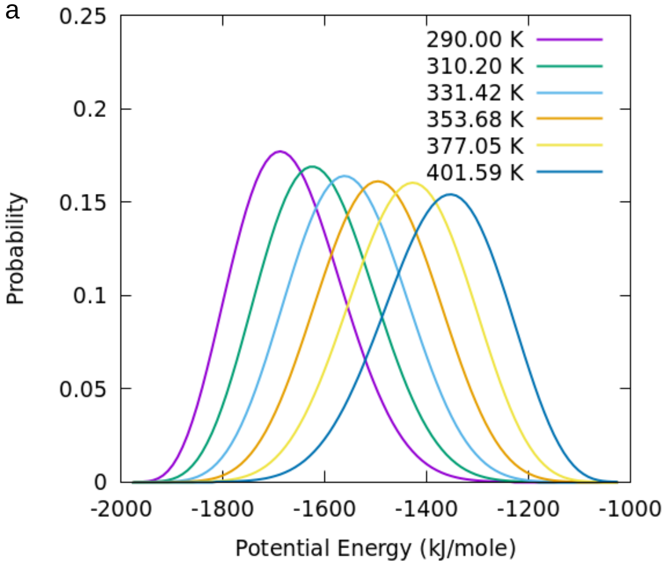

b

Average exchange probabilities:

Replica	0	1	2	3	4	5
Prob.	.36	.38	.39	.40	.40	

Supplementary Figure 3. *REMD energy distributions for wt peptide at 6 different temperatures. Sampling gives very good energy overlaps with exchange probabilities varying from 0.36 to 0.40.*

We summarize folding simulation results in TableS1 for the two independent folding simulation methods. The superscript "1" is used for equilibrium multiple fast-folding simulations while "2" represents REMD results. Random coil and hairpin probabilities, rankings, as well as the experimental $T_{1/2}$ values (from Ref. 26 in the Main Text) are given. Mutant sequences are shown with mutation sites underlined. In correlating simulation results with the experiment, random-coil/hairpin probabilities were compared to experimental aggregation speeds (=$1/T_{1/2}$) while rankings were compared directly to each others..

Table S1: *Folding simulation results (exp=experimental, ran=random-coil, and tot=total hairpin content)*

Mutant	Sequence	$T_{1/2}$	R_{exp}	P^1_{ran}	R^1_{ran}	P^1_{even}	P^1_{odd}	P^1_{tot}	R^1_{tot}	P^1_{xHAT}(x10^{-5})	R^1_{xHAT}	P^2_{ran}	R^2_{ran}	P^2_{even}	P^2_{odd}	P^2_{tot}	R^2_{tot}	P^2_{xHAT}(x10^{-5})	R^2_{xHAT}
LI-QV-KS	ENIKHVLGGGSVQIVYK	3.9	1	0.471	17	0.061	0.059	0.120	1	22.17	1	0.144	15	0.054	0.223	0.278	4	66.08	2
P301S	DNIKHVSGGGSVQIVYK	4.1	2	0.497	15	0.052	0.058	0.110	3	15.74	5	0.188	10	0.069	0.127	0.197	9	61.10	3
P301L	DNIKHVLGGGSVQIVYK	7.2	3	0.467	18	0.059	0.050	0.109	4	17.36	3	0.125	21	0.058	0.151	0.208	8	50.14	4
ED-LI-KS	DNIKHQLGGGSVQIVYK	9.2	4	0.502	13	0.051	0.054	0.105	6	13.88	7	0.273	2	0.033	0.081	0.115	13	8.99	10
ED-LI-QV	DNIKHVLGGGKVQIVYK	9.2	5	0.519	4	0.048	0.050	0.097	9	11.33	9	0.139	16	0.032	0.147	0.180	11	15.19	8
WPL	WDNIKHVLGGGSVQIVYK	13.0	6	0.460	20	0.058	0.048	0.106	5	16.15	4	0.151	14	0.027	0.085	0.112	14	6.15	11
ED-QV-KS	DNLKHVLGGGSVQIVYK	14.1	7	0.466	19	0.055	0.045	0.100	8	13.59	8	0.129	20	0.029	0.184	0.213	7	15.11	9
QV-KS	ENLKHVLGGGSVQIVYK	23.4	8	0.486	16	0.047	0.041	0.089	11	9.26	10	0.137	18	0.043	0.274	0.317	2	49.95	5
LI-KS	ENIKHQLGGGSVQIVYK	29.9	9	0.511	9	0.058	0.054	0.112	2	18.04	2	0.208	9	0.051	0.165	0.216	6	42.50	6
PLW	DNIKHVLGGGSVQIVYKW	30.0	10	0.413	21	0.048	0.038	0.086	12	8.88	11	0.091	22	0.037	0.043	0.080	16	5.79	12
d296	DIKHVPGGGSVQIVYK	31.9	11	0.514	7	0.027	0.052	0.078	16	3.65	17	0.171	12	0.010	0.315	0.325	1	2.99	16
G303V	DNIKHVPGVGSVQIVYK	32.1	12	0.501	14	0.023	0.042	0.065	20	2.24	20	0.236	4	0.008	0.042	0.050	21	0.29	21
ED-QV	DNLKHVLGGGKVQIVYK	38.1	13	0.524	2	0.043	0.041	0.084	14	7.72	13	0.133	19	0.014	0.208	0.222	5	4.31	13
S305N	DNIKHVPGGGNVQIVYK	41.2	14	0.512	8	0.022	0.037	0.058	21	1.74	21	0.245	3	0.023	0.040	0.064	20	2.20	20
LI-QV	ENIKHVLGGGKVQIVYK	55.7	15	0.507	12	0.052	0.051	0.103	7	14.00	6	0.160	13	0.060	0.242	0.302	3	87.08	1
KS	ENLKHQLGGGSVQIVYK	63.1	16	0.508	11	0.045	0.040	0.085	13	8.20	12	0.229	5	0.040	0.150	0.190	10	24.20	7
ED-LI	DNIKHQLGGGKVQIVYK	65.3	17	0.546	1	0.033	0.056	0.089	10	6.08	15	0.294	1	0.021	0.088	0.109	15	4.06	14
WPLW	WDNIKHVLGGGSVQIVYKW	67.5	18	0.401	22	0.043	0.032	0.075	18	5.95	16	0.138	17	0.024	0.045	0.069	19	2.56	17
WT-cis	DNIKHVP-cisGGGSVQIVYK	75.0	19	0.518	5	0.016	0.020	0.035	22	0.48	22	0.223	6	0.002	0.009	0.012	22	0.005	22
V300I	DNIKHIPGGGSVQIVYK	77.8	20	0.514	6	0.026	0.045	0.071	19	3.08	19	0.182	11	0.024	0.053	0.077	18	3.10	15
ED-KS	DNLKHQLGGGSVQIVYK	88.0	21	0.520	3	0.041	0.037	0.078	15	6.31	14	0.215	7	0.014	0.138	0.151	12	2.55	18
WT	DNIKHVPGGGSVQIVYK	—	22	0.508	10	0.025	0.052	0.077	17	3.19	18	0.211	8	0.020	0.060	0.079	17	2.30	19
	$r_{(Pearson-Correlation)}$ with Exp.			-0.18	**-0.47**	0.57	0.56	0.65	0.76	0.72	**0.76**	-0.23	**-0.37**	0.63	0.21	0.26	0.47	0.55	**0.67**

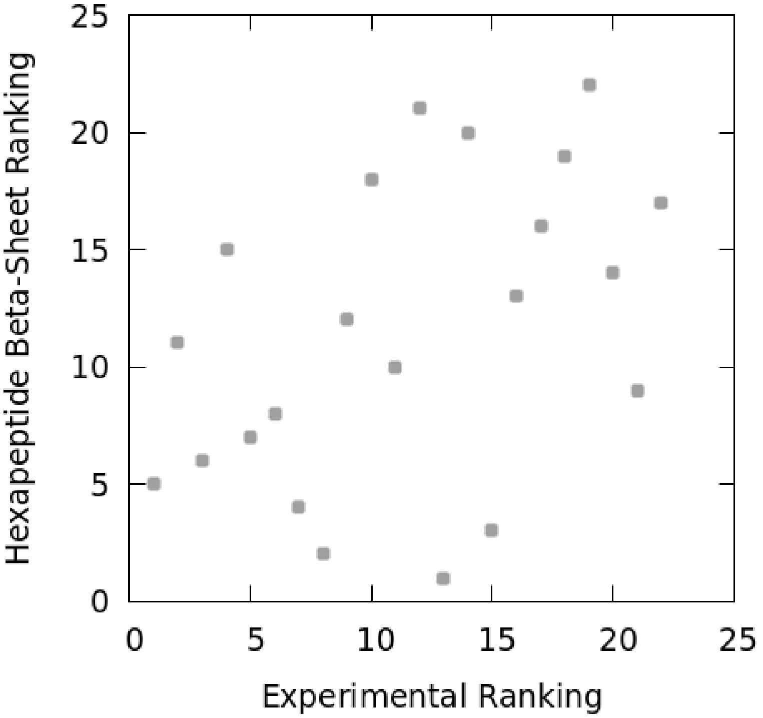

Supplementary Figure 4. *Ranking based on beta-sheet content of the PHF6 hexapepeide segment based on per-residue secondary structure assignments from STRIDE program*

Supplementary Note 4. Dominant Hairpin Folds

Both equilibrium and REMD simulations reveal that the fastest aggregating mutants predominantly fold into hairpins with two different registries. The first one is a perfectly complete hairpin in which all C terminal hexapeptide residues pair with N terminal residues, with 301-305, 299-307, 297-309, and 295-311 innternal hydrogen bondings. Even the two terminal residues (295D 311K) show high stabilities with both backbone hydrogen bonding and side chain salt bridge interactions. The turn region is provided by the three glycines (302, 303, 304). In addition to being complete, this specific hairpin fold brings many favorable amino acid pairings as shown in Supplementary Fig. 5.The second most populated hairpin is two residues shifted one, from the first, with 301-307, 299-309, 297-311 pairings and a turn region over 303G to 305S. Note that two residue shift in registry brings back the same side chains on the same face but produces different pairings (one residue shift, however, would flip the c terminal side chains; e.g. compare 299-307 to 299-308) Therefore, both the first and the second dominant hairpins we see in faster aggregating mutants have the same side-chain directions. We observe that one or both of these hairpins dominate(s) the hairpin ensembles of 15 (out of 22) mutants, with varying probabilities. Because both of these hairpins put the residue 301 in the N-terminal beta-strand side, with its backbone internally hydrogen bonded to the C-terminal side, a proline residue is strongly disfavored at position 301. That is why WT (as well as S305N, G303V, and V300I having 301P) never produced such long hairpins. Instead, we see two other hairpins to dominate the ensembles with varying probabilities.In the first one, with a 5.51% probability in WT, the turn region starts with hydrogen bonding of 302G to 305S, with subsequent 300V-307Q, 298K-309V, and 296N-311K downstream pairings. Proline at 301 sits on the i-1 position (as i=300, the start of the turn) with a solvent-exposed backbone. This hairpin registry does not bring any hydrophobic pairing, while partly stabilized by 295D-311K electrostatic interactions and two glycines being on the turn region (303G and 304G). In the second WT fold, with 4.74 % probability, P301 sits on the i+1 position in the turn region, which is known to be the preferred position for proline (Refs. 42,43 in Main Tex). However, it again produces hydrophobic to charged/polar

pairings in the downstream part (306V-298K, 308I-296N, and 309V-295D), mainly due to the positional restraint brought by proline. Because this WT fold leaves both 310Y and 311K completely unpaired in the hexapeptide, and further 309V does not interact well with the corresponding 295D on the N-terminal side not having a proper beta-sheet form, this specific fold cannot meet our hairpin criteria requiring the whole hexapeptide other than the mobile terminal 311K must be in beta-sheet. Therefore, this fold is not counted as a hairpin and is excluded for all mutants.

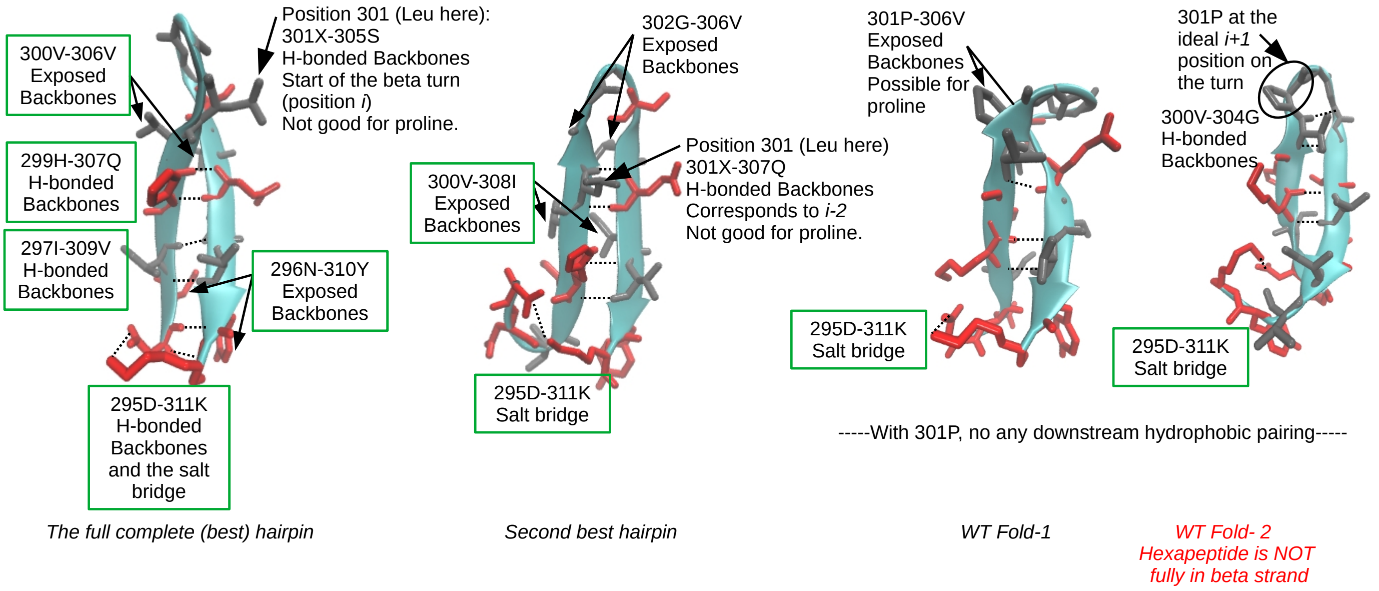

LI-QV-KS:	17.88%	2.62%	1.79%	0.85%
P301L:	7.05%	6.31%	1.52%	0.04%
P301S:	5.06%	5.97%	1.66%	1.42%
WT:	0.04%	0%	5.51%	4.74%

Supplementary Figure 5. *The two dominant hairpin folds for the fast aggregating mutants as well as those for WT. Hydrophobic residues (P, G, V, I, L) are in gray while polar/charged ones (D, N, K, H, S, Q, Y) are in red. The chemically preferred amino acid pairings are shown in green boxes. PHF6 hexapeptide residues are on the beta-sheet for the first three hairpins while the last fold leaves 310Y and 311K completely unpaired and 309V not having a proper beta-sheet structure due to bad pairing with the n-terminal 295D.*

Supplementary Note 5. Cross-beta transitions in explicit solvent equilibrium MD simulations

Supplementary Fig.6 shows the simulation results of trimeric hairpin systems. Four independent simulations each with 1 μs (a total of 4 μs sampling) were performed for each system. These equilibrium simulations are with explicit solvent molecules, with no bias/force, as explained in the method section. As seen in the figure, dimeric setups did not show any fibril-like cross-beta structures while only the trimer with a shifted hairpin exhibited such transitions.

Supplementary Note 6. Topological argument for why we need a registry shifted hairpin for parallel cross-beta transition

An important property of amyloid fibril structures is that hydrophobic residues are protected from solvent while polar/charged residues are exposed, as expected. Here, we show that three identical hairpins having one side more hydrophobic than the other side, cannot simultaneously protect all the 3 hydrophobic faces from the solvent when there must be a parallel cross-beta transition. An anti-parallel cross-beta transition is topologically possible, although associated with large free energy barriers as we showed. However, when a hairpin shifts its registry, from i-j internal hydrogen bonding to i-(j+-1) bonding, then the trimeric complex can make fibril-like parallel cross beta transition (Supplementary Fig.7).

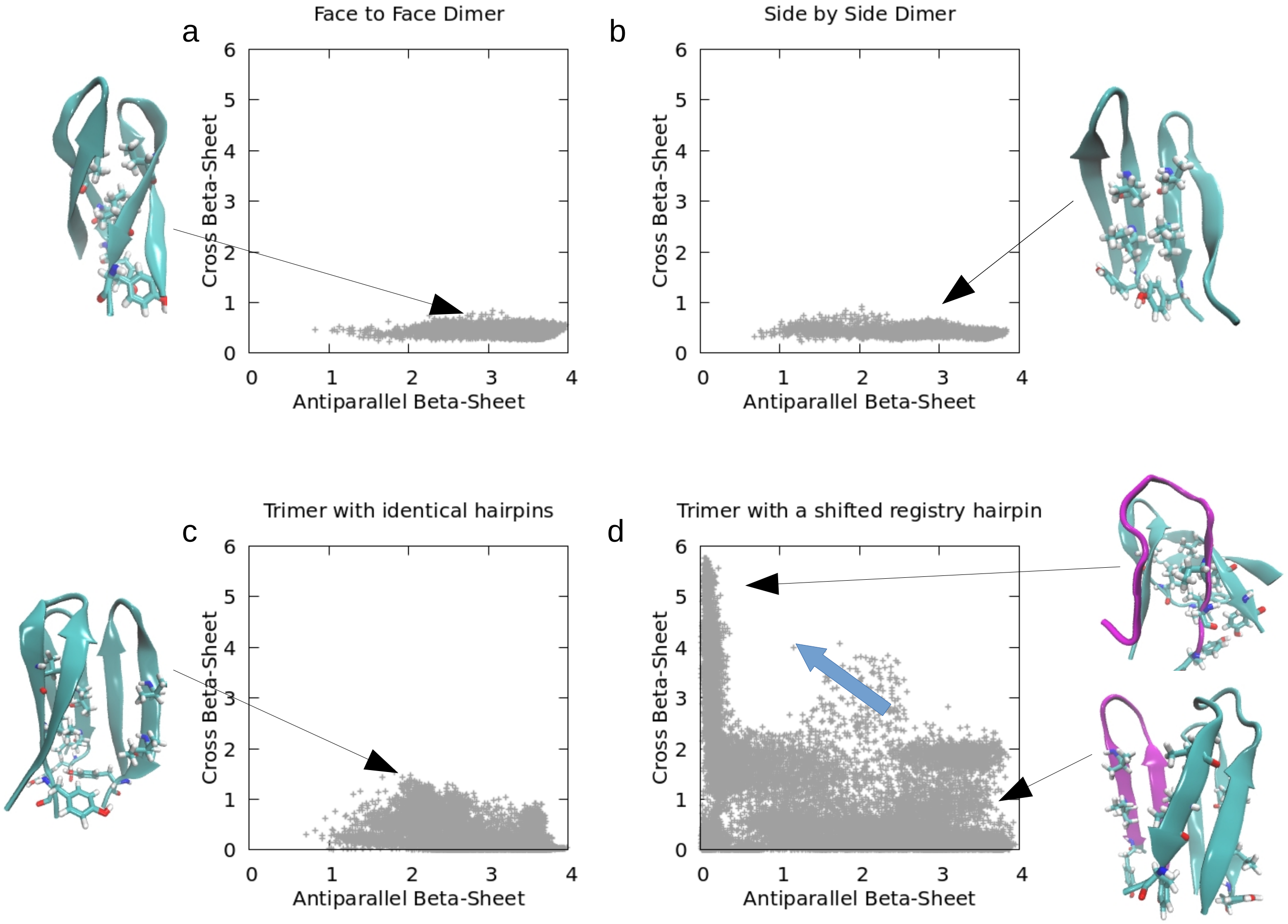

Supplementary Figure 6. *Cross beta transitions in equilibrium dimer and trimer simulations. Only the trimer with a shifted registry hairpin showed the transition. In both dimer and trimer plots, x and y values are normalized per hairpin*

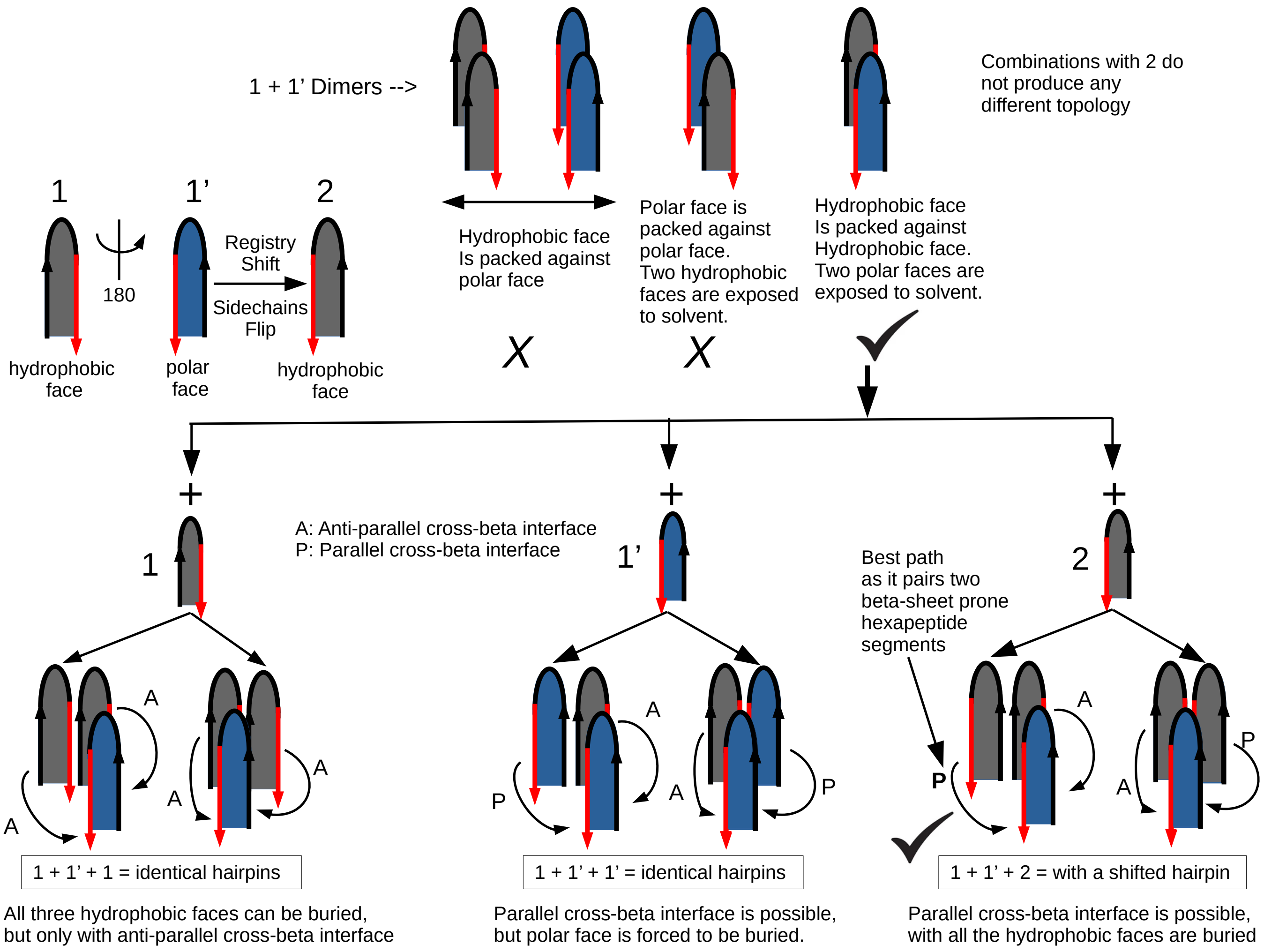

Supplementary Figure 7. *Topological demonstration that a shifted registry hairpin is required in order to get a cross-beta transition that buries all three hydrophobic faces of hairpins.*

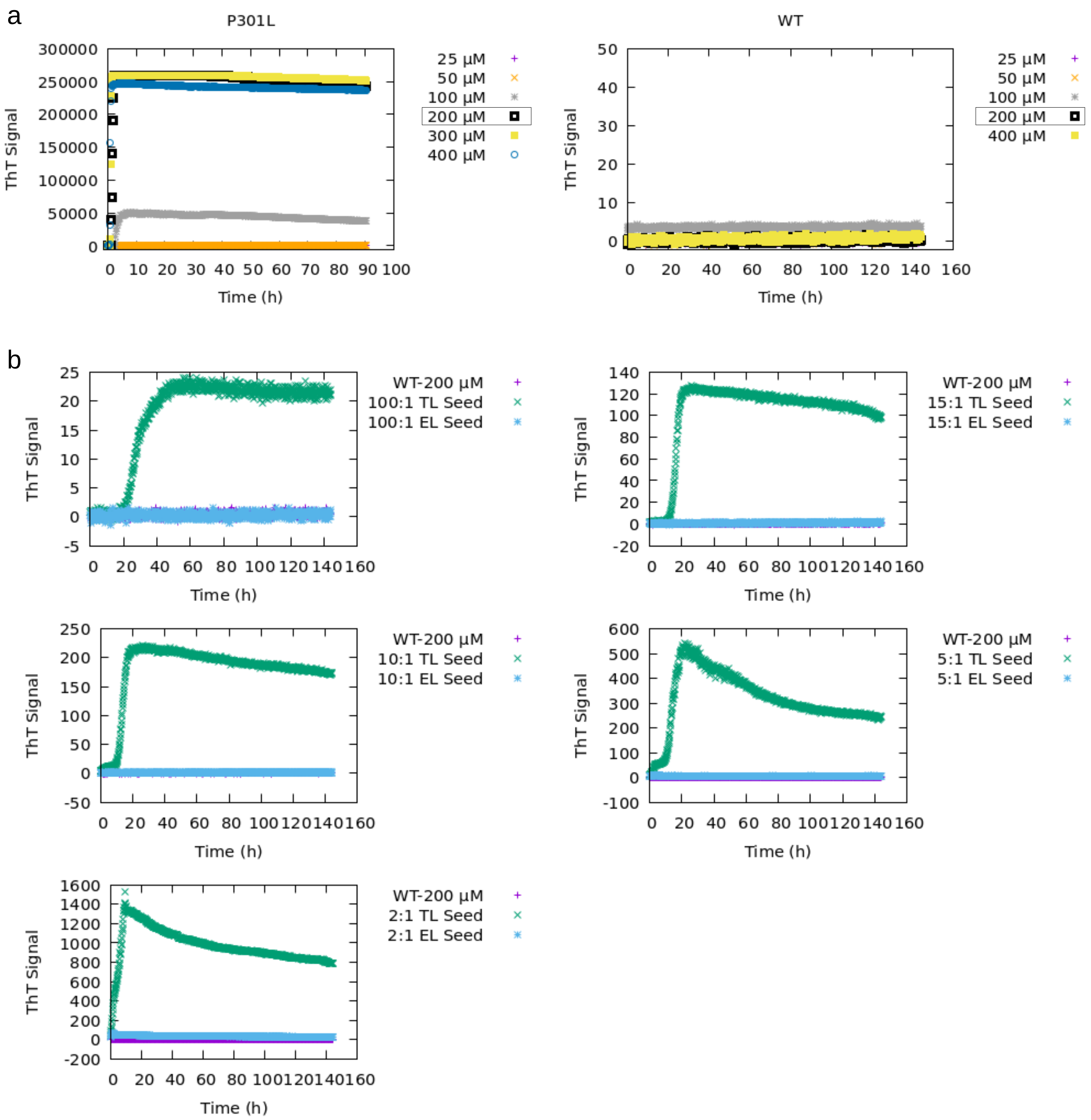

Supplementary Figure 8. *Concentration dependence of WT aggregation by TL and EL seeds. a, Establishing aggregation capacity of peptides using aggregation prone P301L mutant. 200 µM is concentration at which we see a jump in fluorescence intensity. Pure WT peptide do not show any ThT signal at any concentration. b, Seeding of 200 µM WT peptide with TL and EL dimers at different concentration ratios. In all, TL dimer robustly seeds WT fibrils while EL dimer does not. Data are presented as averages of four replicates.*

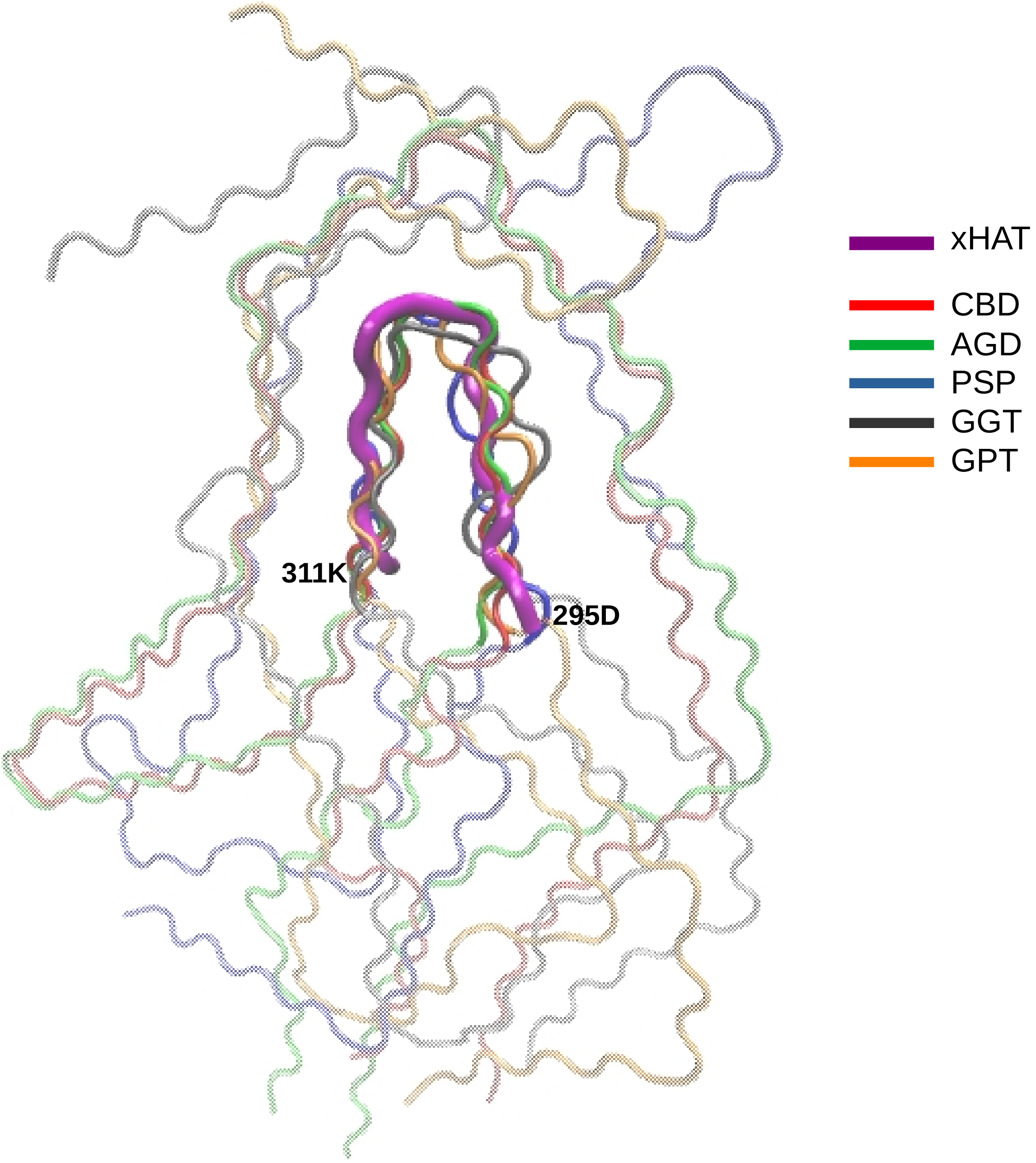

Supplementary Figure 9. *Superposition of cross-beta monomeric peptide in xHAT model (thick purple) with different fibril structures of 4R tauopathies. The overlapping sequence 295-311 is shown in solid colors while the rest of the repeat domain is shown in semi transparency. The naming and associated structures are as follows: CBD (Corticobasal Degeneration, 6tjo.pdb), AGD (Argyrophilic Grain Disease, 7p6d.pdb), PSP (Progressive Supranuclear Palsy, GGT (Globular Glial Tauopathy, 7p66.pdb), GPT (GGT-PSP-Tau, 7p6a.pdb). Note that for some tauopathies like AD (Alzheimer's Disease), PiD (Pick's Disease), and CTE (Chronic Traumatic Encephalopathy), the 295-311 sequence segment is missing as they are not associated with 4R tau isoform (see Reference 48 in the Main text for structure-based classification of tauopathies).*

Supplementary References

[S1] Andrea G Cochran, Nicholas J Skelton, and Melissa A Starovasnik. "Tryptophan zippers: Stable, monomeric b-hairpins". In: Proceedings of the National Academy of Sciences 98.10 (2001), pp. 5578–5583.

[S2] Xavier Daura et al. "Peptide folding: when simulation meets experiment". In: Angewandte Chemie International Edition 38.1-2 (1999), pp. 236–240.
